# Comparison of proximal and distal laparoscopic ureteroureterostomy for complete duplex kidneys in children

**DOI:** 10.1007/s11255-024-04108-3

**Published:** 2024-06-11

**Authors:** Xiao-Jiang Zhu, Li-Qu Huang, Saisai Liu, Jun Dong, Hao-Bo Zhu, Chen-Jun Chen, Li-Xia Wang, Yun-Fei Guo, Yong-Ji Deng, Ru-Gang Lu

**Affiliations:** Department of Urology, Children’s Hospital of Nanjing Medical University, 72 Guangzhou Road, Nanjing, 210008 China

**Keywords:** Child, Complete duplex kidney, Laparoscope, Ureteroureterostomy

## Abstract

**Objective:**

To compare efficacy of proximal and distal laparoscopic ureteroureterostomy (UU) for complete duplex kidneys in children.

**Methods:**

Patients who underwent laparoscopic UU for complete duplex kidneys between December 2016 and July 2022 were reviewed retrospectively. 71 patients who had normal lower pole moiety without vesicoureteral reflux (VUR) were recruited. All of them underwent ultrasound, voiding cystourethrography (VCUG), renal scintigraphy, and magnetic resonance urography preoperatively. Proximal laparoscopic UU was performed in 35 patients and distal laparoscopic UU in 36 patients. Double J stents were placed in normal lower pole moieties. Clinical data, including general information, diagnosis, surgical management, imaging characteristics, clinical symptoms and postoperative complications (classified according to the modified Clavien–Dindo classification), and length of stay were recorded. Measurement date comparisons between groups were performed by *t* test, counting date were analyzed by chi-square test*.*

**Results:**

The study consisted of 71 patients (56 females and 15 males) with complete duplex kidneys (41 in left kidney and 30 in right kidney). The patients’ mean age was 34 m (range 3–161 m) and follow-up ranged from 25 to 81 m. No significant difference was found in age and follow-up time between the two groups. Laparoscopic UU was performed in all patients successfully. The operation time of the two groups was 108.42 ± 26.95 min for distal UU vs 121.46 ± 35.15 min for proximal UU(*p* = 0.14). No significant difference in postoperative complications was seen between the two groups (22.2% vs 31.4%, *p* = 0.345). However, in terms of the grading of postoperative complications, the proximal UU group had a higher grade (3 of them had a grade of IV) and more serious complications.

**Conclusions:**

There was no significant difference in the overall incidence of complications between distal and proximal UU. Compared with proximal laparoscopic UU, distal laparoscopic UU is easier to perform with less injury to the peripheral tissues. Postoperative complications of proximal UU are more serious and more difficult to manage. We recommend complete duplex kidney ureteral reconstruction with distal UU.

## Introduction

Duplex kidney, a congenital anomaly of the urinary system, has a reported prevalence of 0.8% among children [1]. The various anomalies accompanying duplex kidney cause substantial changes in renal function. Therefore, individualized therapies are needed for children with duplex kidneys, which pose challenges for pediatric urologists [2]. The management of hydronephrosis and ureterectasia in patients with duplex kidneys is controversial. In the past, most urologists chose heminephrectomy and ureterectomy to manage duplex kidneys. In recent years, laparoscopic UU is being increasingly performed due to technological advances. Compared with heminephrectomy, laparoscopic UU confers a lower surgical risk and less damage to normal renal tissues, and it has no obvious impact on the morphology or function of the normal lower pole moiety [3–5]. Based on the abovementioned advantages, UU has gradually been accepted by pediatric urologists as an effective strategy for managing complete duplex kidneys.

When used to treat a complete duplex kidney, UU has two approaches: the proximal approach and the distal approach. Some researchers believe that the proximal approach should be used in laparoscopic UU, and that the distal approach should be adopted in open surgery [6]. To the best of our knowledge, there are no detailed comparisons of the two approaches. In the present study, we retrospectively reviewed the clinical data of 71 UU patients and compared the outcomes of the two approaches.

## Methods

The indications for UU are a complete duplex kidney with a normal lower pole and ureter, no VUR from the lower pole system. The presence of ureterocele and/or ectopy of the upper pole ureter does not affect the prognosis. If the function of the upper pole measured by renal scintigraphy is not poor, we recommend UU; if the function is relatively poor, we will provide patients with a full explanation of the advantages and disadvantages of these different surgeries and they can choose between UU and heminephrectomy. Informed consent was obtained from the patients’ guardians before surgery. This study was approved by the Medical Ethical Committee of the Children’s Hospital of Nanjing Medical University.

A total of 71 children with complete duplex kidney who underwent UU to treat upper moiety hydronephrosis and ureterectasia in the Urology Department of Children’s Hospital of Nanjing Medical University from December 2016 to July 2022 were recruited. The 71 patients were divided into two groups based on the surgical approach: the proximal UU group and the distal UU group. All of them underwent urologic ultrasound, magnetic resonance urography (MRU), and voiding cystourethrography (VCUG) preoperatively, and some of them underwent renal scintigraphy.

Proximal UU was performed as follows: under general anesthesia, the trocars were placed in the same manner as in laparoscopic pyeloplasty. The traction thread was stitched to the upper pole pelvis and the two ureters and then traversed out of the body. Below the horizontal thread stitched to the lower moiety, a cut was made to sever the distended upper pole ureter. Then an opening (approximately 1/3 of the circumference) was made in the lower pole ureter to cleave the ureter longitudinally from the distal to the proximal end for approximately 1.5 cm. From the opening, the double J stent was placed in the lower moiety. Using 5–0 absorbing sutures, both ureters were anastomosed and forming a Y shape. The distal part of the upper pole ureter was dissociated downward as much as possible, excised, and ligated. Finally, routine abdominal drainage was performed and the abdominal incision was closed.

The distal UU was performed as follows: The trocars were inserted through the umbilicus and two lateral sites. To expose the dilated upper pole ureter and the normal lower pole ureter, an incision was made in the peritoneum above the ovary/the vas deferens. The lower pole ureter, vas deferens/ovary and ovarian duct were kept intact. In the pelvic cavity, the dilated upper pole ureter was mobilized downward and excised as distally as possible and the stump was ligated. For female patients, the ureter was mobilized around the uterus, under the ovarian duct, above the round ligament and as close as possible to the bladder. At the entry site of the pelvic cavity, the upper pole ureteric stump was excised. Then the anterior wall of the lower pole ureter was vertically cut to approximately 1.5 cm. The side of the lower pole ureter was anastomosed with the resected end of the upper pole ureter using 5–0 absorbing sutures. A double J stent was placed in the lower pole of the pelvis and ureter. The remaining steps were performed as described in the proximal group.

**Fig. 1:**
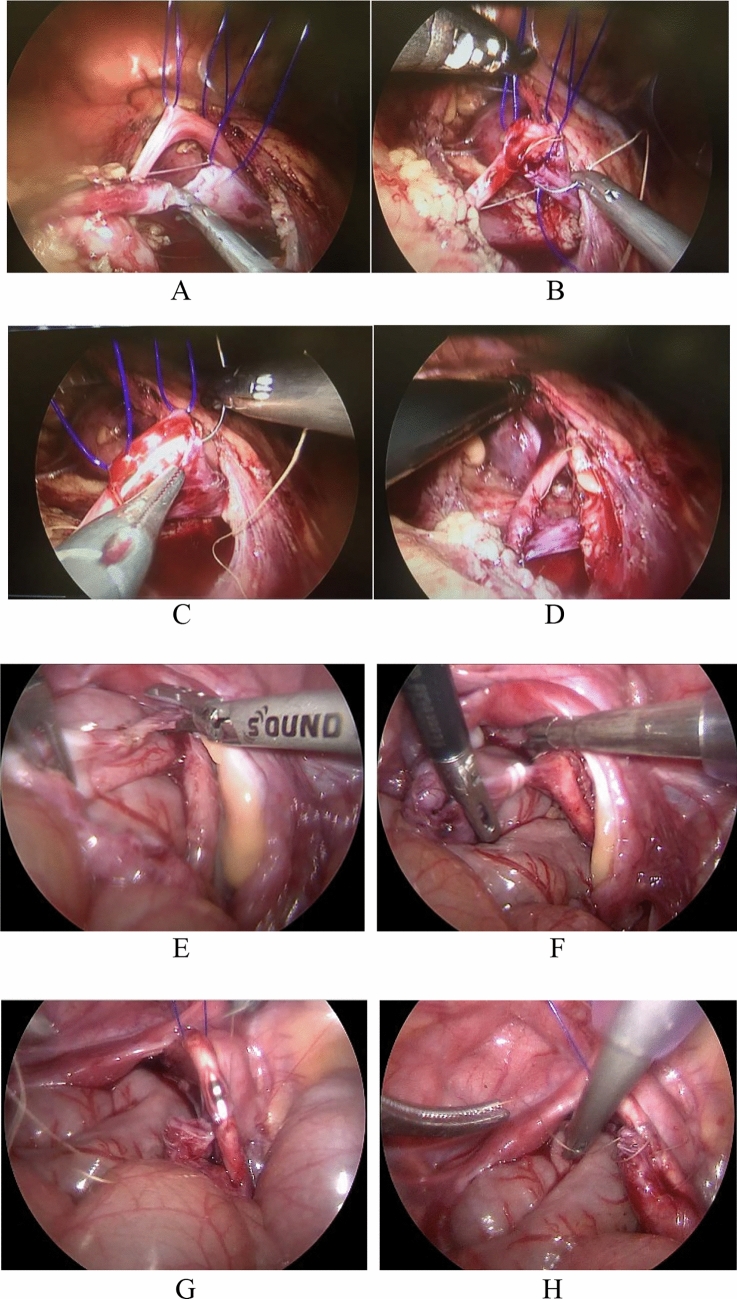
Proximal UU: (**A**). Double ureteral suspension, disconnection of upper half kidney ureter (**B**–**C**). Horizontal anastomosis of lower pole of kidney with double ureters (**D**), ureteral anastomosis completed. distal UU: (**E**). Pelvic level free two duplicate ureters (**F**). Ligation of duplicate ureters at the bladder distal (**G**–**H**). Pelvic anastomosis of two ureters

The key steps of proximal and distal UU surgery are shown in the following Fig. 1:

The same postoperative management strategy was applied in both groups. The drainage tube was removed 2–3 days after surgery. The double J stent was removed 1–2 months after surgery. Radiological assessments such as ultrasound were performed regularly postoperatively.

The data of the two groups were compared using two-tailed tests, and the categorical variables were compared using chi-square analysis. All the data were processed using Prism 6 software (GraphPad, San Diego, CA). A *p* value of 0.05 was used as the cutoff for statistical significance.

## Results

No statistically significant differences were found in age, sex, symptomatic/asymptomatic status, or left/right kidney status between the two groups. (Table 1). Proximal UU was performed in 35 kidneys (19 left, 16 right) of 35 patients (6 males, 29 females) with a mean age of 35.6 months. Preoperatively, 19 patients presented clinical symptoms, such as abdominal pain, urinary tract infection and urinary incontinence between two urine passages, and 16 had no symptoms. Seven patients had concurrent VUR in the upper pole moiety, 17 had concurrent ureterocele, four had concurrent ureteral ectopic opening and six had mild pyeloneal separation in the lower pole moiety. Distal UU was performed in 36 patients (9 males and 27 females, 22 left kidneys and 14 right kidneys) with a mean age of 31.2 months. Preoperatively, 21 patients were symptomatic and 15 were asymptomatic. Twelve patients had concurrent ureterocele, 7 had ureteral ectopic opening and ten had mild pyeloneal separation in the lower pole (Table 2).

**Table 1:** Demographics of patients with proximal laparoscopic ureteroureterostomy and distal laparoscopic ureteroureterostomy.

	Distal UU (*n* = 36)	Proximal UU (*n* = 35)	*P* value
Age (m)	31.8 ± 41.73	35.63 ± 29.79	0.096
Gender			
Male	9(25%)	6(17.1%)	0.453
Female	27(75%)	29(82.9%)	
Laterality			
Left	22(61.1%)	19(54.3%)	0.658
Right	14(38.9%)	16(45.7%)	

*p* values represent statistical comparison of proximal laparoscopic ureteroureterostomy as a whole versus distal laparoscopic ureteroureterostomy as a whole.

**Table 2:** Comparison anatomical and preoperative variables in patients undergoing proximal laparoscopic ureteroureterostomy and distal laparoscopic ureteroureterostomy

	Distal UU (*n* = 36)	Proximal UU (*n* = 35)	*P *value
Ureterocele present?	12 (33.3%)	17 (48.6%)	0.248
Ectopic insertion?	7 (19.4%)	4 (11.4%)	0.377
Preop Vesicoureteral reflux(Upper pole)	6 (16.7%)	7 (20%)	0.908
Lower pole hydronephrosis			
none	26 (72.3%)	29 (82.9%)	0.265
Mild hydronephrosis of the lower pole (no indication of surgery)	10(27.8%)	6(17.1%)	
No clinical symptoms/pregnancy test	15(41.7%)	16 (45.7%)	0.658
With clinical symptoms			
Preoperative Febrile UTIs	13 (36.1%)	11 (31.4%)	
Urinary incontinence/leakage of urine	6 (16.7%)	4 (11.4%)	
Abdominal pain/vomit	2 (5.6%)	4 (11.4%)	

*p* values represent statistical comparison of proximal laparoscopic ureteroureterostomy as a whole versus distal laparoscopic ureteroureterostomy as a whole

UU was performed in all patients successfully with no conversion to open surgery. The proximal group had a mean surgery time of 121.46 min, and a 25- to 81-month follow-up. The distal group had a mean surgery time of 108.42 min. The follow-up time ranged from 15 to 71 m. In the proximal group, 5 children developed febrile urinary system infections after the operation, one patient had residual ureteral infection and recurrent urinary tract infection for one year. During the follow-up period, 2 children had exacerbated hydronephrosis, without other discomfort, and 1 patient had hydronephrosis aggravated by febrile UTI due to anastomotic stenosis, the hydronephrosis was relieved after nephrostomy. Another patient experienced severe complications due to anastomotic stricture after the operation. Nephrostomy and pyeloureteroplasty were performed many times. At present, the patient have recovered well and are still under close follow-up observation. One case of urogenic peritonitis was caused by urinary leakage of the anastomotic fistula after proximal UU. Nephrostomy was performed first in emergencies, followed by a secondary UU.

In the distal group, 5 children experienced febrile urinary system infection after the operation, 2 patients experienced aggravated hydronephrosis without other discomfort during the follow-up period, and one patient experienced new lower pole vesicoureteral reflux with febrile UTI after the operation. The child was cured after bladder and ureter replantation. One child suffered from abdominal pain due to blockage of the double J tube after the operation, and hydronephrosis was aggravated, which improved after double J tube replacement.

All other patients showed improvement in terms of hydroureterosis and smooth ureteral drainage in the upper pole moiety, as shown by postoperative urologic ultrasound and renal scintigraphy. In addition, during the follow-up period, no hydronephrosis, hydroureterosis, or ureterectasia was found in the lower pole moiety (ADP < 1 cm). The preoperative symptoms were completely resolved. The surgical and follow-up data are shown in Table 3.

**Table 3:** Postoperative outcomes of patients undergoing proximal laparoscopic ureteroureterostomy and distal laparoscopic ureteroureterostomy

	Distal UU (*n* = 36)	Proximal UU (*n* = 35)	*p *value
Operation time(min)	108.42 ± 26.95	121.46 ± 35.15	0.14
Length of stay(d)	10.94 ± 2.63	12.31 ± 6.87	0.917
Total complications	8(22.2%)	11(31.4%)	0.345
Febrile or afebrile UTIs after initial postoperative period	5(13.9%)	5(14.3%)	0.345
Infection of stump	0	1(2.9%)	
Postoperative hydronephrosis aggravated	2(5.6%)	2(5.7%)	
Re-operation	2(5.6%)	3(8.6%)	
Complications Clavien–Dindo grade			
1	3(8.3%)	3(8.6%)	–
2	3(8.3%)	3(8.6%)	
3	2(5.6%)	2(8.6%)	
4	0	3(8.6%)	
5	0	0	

*p* values represent statistical comparison of proximal laparoscopic ureteroureterostomy as a whole versus distal laparoscopic ureteroureterostomy as a whole

## Discussion

Currently, no consensus has been reached on the management of complete duplex kidney with concurrent hydronephrosis in the poorly functioning upper pole moiety. At the beginning of the last century, Foley first reported UU as a treatment for duplex kidney [7]. However, for quite a long time, upper pole heminephroureterectomy was the most commonly used management method for this disease. Preserving the poorly functioning upper moiety is believed to increase the risk of hypertension, albuminuria, or even cancer, and ureteroureterostomy might exert adverse effects on the normal lower pole moiety. Due to advances in surgical techniques and an in-depth understanding of UU, in recent years, UU has been gradually adopted by an increasing number of urologists to treat complete duplex kidneys. Accumulating evidence has shown that UU has no harmful impact on the lower moiety and that preserving the upper moiety does not increase the occurrence of hypertension or other diseases. Hence, UU has been acknowledged as a safe and effective treatment for complete duplex kidney [4, 8–10].

The use of laparoscopy decreases the rate of open pediatric urologic surgeries, however, robotic surgery is still not widely applied, especially in developing countries. Therefore, laparoscopic UU has been a common choice for the treatment of complete duplex kidney. Laparoscopic UU involves proximal and distal approaches involving the lower pole moiety. Both approaches have been reported to have favorable outcomes and obvious advantages when compared with upper pole heminephroureterectomy. However, which approach is better remains controversial. Chandrasekharam V. et al. [11] reported that the distal approach causes less damage to peripheral tissues and is associated with a lower incidence of postoperative complications than the proximal approach. Storm et al. [12] used the proximal approach and reported favorable surgical outcomes. Thus, the surgical approach should be selected based on the surgeon’s preference and experience. To the best of our knowledge, few studies have compared the two approaches in detail. In the present study, we compared the complexity, peripheral tissue damage and postoperative complications of the two approaches, hoping to provide clinical evidence of laparoscopic UU for the treatment of complete duplex kidney. In our study cohort, there was no significant difference in the overall incidence of complications between the distal UU and proximal UU groups [(8/36) 22% vs. 11/35(31%), *P* = 0.345]. However, in terms of postoperative complication grades, the proximal UU group had higher grade complications (3 of them had a grade of IV complication). In addition, 2 children in the proximal UU group had serious complications due to anastomotic stenosis, 1 child had ureteral stump infection, and no patient in the distal UU group had anastomotic stenosis or stump infection. There was no difference between the two groups in terms of postoperative urinary system infection, aggravation of hydronephrosis and reoperation rate.

The key points of the proximal approach are as follows: First, the ureter of the upper moiety was horizontally severed at the lower pole of the kidney. Second, the lower moiety of the ureter was dissociated, and a vertical incision of approximately 1 cm was made. The incision should be carefully made so as to avoid severing the normal lower moiety of the ureter. Third, end-to-side anastomosis of the severed upper ureter with the lower ureter was performed. Next, the double J stent was placed. Finally, the dilated upper ureter was dissociated as low as possible and severed. Compared to upper pole heminephrectomy, this approach has distinct advantages. It eliminates the possible complications caused by upper pole heminephrectomy, such as wound bleeding, urinary extravasation and damage to the normal renal tissues in the lower moiety (which may even result in lower pole heminephrectomy) [4, 6].

In our research, proximal UU was less frequently recommended than distal UU due to surgical procedure and risks. In the distal approach, it is easier to dissociate the distal ureter due to its superficial position. Furthermore, a considerable amount of operational space is available. Additionally, the distal approach prevents the dissociation of the colon and thus does not have an impact on gonadal arteries. In addition, there is no need to remove much of the upper ureter; therefore, the damage to the lower ureter and the impact on the ureteral blood supply can be reduced [11]. Furthermore, the distal approach has less impact on the gastrointestinal tract, which ensures a quick recovery. In this study, the operation times of the two groups were 108.42 ± 26.95 min for the distal UU group and 121.46 ± 35.15 min for the proximal UU group. However, if the cystoscopic detection and retrograde intubation times were excluded, the distal group would have a shorter surgery time. The key points of the distal approach are as follows: (1) maintain the integrity of the gonad, gonadal arteries and ureter; (2) and excise the upper pole ureter stump as close to the bladder as possible. (3) ensure the length of the lower ureter incision is the same as the diameter of the upper ureter. If the two does not match, the upper ureter incision must be modified to ensure an open anastomotic stoma. (4) The dissociation of the lower ureter should be minimized to eliminate the impact on the ureteral blood supply and the consequent anastomotic stenosis. (5) During surgery, clamping or gripping should be avoided to minimize the risk of injury.

No consensus has been reached regarding the approach to total ureteral stump excision of the duplex kidney. Some believe that there is no need for total excision because of the low incidence of stump infection and the potential for uroclepsia or neurogenic bladder caused by bladder neck injury after total excision [11, 13]. Chandrasekharam V. et al. [11] reported favorable outcomes with UU without total ureteral stump excision. However, at the 6-year follow-up visit, De Caluwe et al. [14] reported a 10% rate of reoperation for stump excision due to stump infection. Additionally, Ade-Ajayi et al. [15] reported an 8% rate of reoperation caused by stump infection after upper pole heminephrectomy. In the present study, none of the 36 patients in the distal group had stump infection because the ureteral stump. By was nearly completely excised. In contrast, among the 35 patients in the proximal group, due to incomplete ureteral excision, stump infection occurred in one patient who had concurrent upper pole VUR preoperatively, for an incidence rate of 3%. Based on the above evidence, we believe that near-total excision of the ureteral stump is necessary, especially for patients with concurrent upper pole VUR. Needle total ureteral stump excision decreased the incidence of stump infection in the distal group.

A theoretical concern is poor urine drainage in the upper moiety after treating ureterectasia with the distal approach because this approach may lead to reinfection of the urinary system and abdominal pain. In particular, the weak peristaltic function caused by the maldeveloped upper moiety and ureter may require reoperation. Hence, it is believed that horizontal end-to-side anastomosis of the lower moiety combined with removal of the expanded ureter is a favorable management method. To verify this, we followed up patients in the distal group with obvious upper moiety ureterectasia and found no clinical symptoms such as urinary infection. Postoperative imaging examinations such as ultrasound, revealed apparent relief of hydronephrosis and narrowing of the dilated ureter in the upper moiety. Some patients even showed a normal upper moiety in the examinations.

Another concern from clinicians is complications (such as anastomotic stenosis). Mcleod [9] reported a 2% occurrence of anastomotic stenosis after UU among 43 patients with duplex kidney. There was no anastomotic stenosis in 36 patients who underwent distal UU. In the proximal UU group, there were 2 children with severe complications due to postoperative anastomotic stenosis, and hydronephrosis was aggravated with repeated febrile urinary system infections. Hydronephrosis in these two patients was relieved after two or more operations in the later stage. Despite the low incidence of anastomotic stenosis and other adverse reactions in the lower pole collecting system, effective treatments for postoperative complications are still needed. In the proximal approach, upper moiety removal or another end-to-side anastomosis is needed. In the distal approach, an end-to-side anastomosis at a nearby site or a vesicoureteral replantation is needed. Thus, it is more difficult to treat complications at the anastomotic stoma via the proximal approach than via the distal approach. Some studies have reported yo-yo reflux following ureter anastomosis [13], which has not yet been verified [11, 16]. According to Lashley DB [17], no yo-yo reflux occurred in 100 duplex kidney patients who underwent ureteroureterostomy. During our follow-up examination, except for children with anastomotic stenosis or hydronephrosis, the dilated ureters of all the other patients were relieved, and no yo-yo reflux was found postoperatively.

The objective of this study was to compare the distal approach with the proximal approach for UU. Stringent inclusion criteria were applied to ensure that the patients in both groups shared almost the same manifestations of the disease. All the patients enrolled had complete duplex kidneys with concurrent abnormal upper moieties and ureters, together with normal lower moieties and ureters. Many researchers believe that UU can be performed in the lower moiety with concurrent mild reflux, because the probability of reflux in the lower moiety is significantly higher than that in the upper moiety, and favorable outcomes were also found in follow-up studies [12]. The above understanding warrants further exploration of the indications for UU for a complete duplex kidney.

This study has the following limitations: First, the sample size was not large enough to produce more convincing conclusions, which is also a limitation of other studies on UU. Second, this was a single-center retrospective study, which may involve selection bias. Third, the follow-up time of a few patients was short (the shortest follow-up time was 15 months).We hope that prospective studies will be designed under the cooperation of multiple institutions to provide more reliable data for clinical practice.

## Conclusions

Compared with the proximal approach, the distal approach has several advantages, such as more operation space for laparoscopy and a lower chance of dissociating the surrounding tissues and organs. Hence, the distal approach is especially recommended for patients with concurrent VUR in the upper pole moiety. In addition, although there was no significant difference in the overall incidence of complications between proximal UU and distal UU surgery, the proximal UU group was prone to more serious complications (e.g., anastomotic stenosis), and because of the limited retention of the upper ureter, it was more difficult to manage the subsequent complications.

## Data Availability

The original data presented in the study are included in the article, further inquiries can be directed to the corresponding author or the first author.
